# The complete chloroplast genome of the ornamental plant *Primula violaris* (Primulaceae)

**DOI:** 10.1080/23802359.2021.2002206

**Published:** 2021-11-29

**Authors:** Weiguo Chai, Huimin Li, Caijuan Zhang, Bo Zhao, Pengguo Xia

**Affiliations:** a Institute of Biotechnology, Hangzhou Academy of Agricultural Sciences, Hangzhou, China; b Key Laboratory of Plant Secondary Metabolism and Regulation of Zhejiang Province, College of Life Sciences and Medicine, Zhejiang Sci-Tech University, Hangzhou, China

**Keywords:** Chloroplast genome, *Primula violaris*, phylogenetic analysis, Primulaceae

## Abstract

*Primula violaris* is a perennial herb distributed throughout the western part of Hubei Province and the southern part of Shaanxi Province, China. We present on of the first reports of the complete chloroplast genome sequence for *P. violaris*. The complete chloroplast genome was 153,630 bp in size, including a large single-copy (LSC) region of 84,526 bp, a small single-copy (SSC) region of 17,812 bp, and a pair of inverted repeat (IR) regions of 25,646 bp. There were 130 genes in the chloroplast genome, including 86 CDSs, 36 transfer RNA (tRNA) genes, and eight rRNA genes. Phylogenetic analysis showed that *P. violaris* is closely related to *P. oreodoxa*. (NC_050848).

Primulaceae is a large family of angiosperms that includes nearly 1000 species, with more than 500 species in the genus Primula of which more than half of them are distributed in China. Primulaceae not only has important scientific significance in the evolution of angiosperms, but also high ornamental value (Chen and Hu 1996). There is still considerable doubt about the phylogeny of Primula, mainly due to frequent interspecific hybridization and gene introgression. *Primula violaris* Smith and Fletcher (1944) is a perennial herb variety of Primulaceae that is distributed in the western part of Hubei Province and the southern part of Shaanxi Province, China. It grows under the hillside forests, ditches and roadsides, and at an altitude of 1000 1500 m (Committee of flora of China 1990). *Primula violaris* was first recorded in 2009 (Liu 2009) and photographed again in 2016 (Zhou 2016). Because of the ornamental value of *P. violaris* it is used to enhance the environment

In this study, fresh leaf tissue was collected from *P. violaris* growing on a mountain at an altitude of 1686 m, in Shiquan County, Ankang City, Shaanxi Province (33°1200″N,108°2004″E). The voucher specimen was preserved at the Herbarium of Xi’an Botanical Garden, http://www.xazwy.com; voucher number: *Xun Lulu* et al. *01376* and xunlulu@xab.ac.cn). Total genomic DNA was extracted using a modified CTAB method (Doyle 1987) and deposited at the Key Laboratory of Plant Secondary Metabolism and Regulation of Zhejiang Province, Zhejiang Sci-Tech University (http://sky.zstu.edu.cn) under the manufacturer number ZSTUX0010 (collected by Pengguo Xia and xpg_xpg@zstu.edu.cn). We constructed a genomic library for Illumina paired-end (PE) sequencing using Illumina Hiseq X Ten sequencer. The software NOVOPlasty v2.7.2 (Dierckxsens et al. 2017) was used to assemble the complete chloroplast genome of *P. violaris* and Geneious Prime (Ammundsen and Duran 2019) and reference to the sequence of *Primula bracteate* (NC_053592), sequence was used to construct the chloroplast genome, which was and submitted it to GenBank (GenBank ID MW970137). The complete *P. violaris* chloroplast genome sequence was 153,630bp in length.with a GC content of 37.1%. It was composed of a large single copy region (LSC) of 84,526bp, a small single copy region (SSC) of 17,812bp and a pair of inverted repeats (IRs) that were 25,646bp in length. This genome contained 130 genes, including 36 transfer RNA (tRNA) genes, eight ribosomal RNA (rRNA) genes, and 86 gene coding sequences (CDS). Among them, all rDNAs were located in the IRs.

Based on the complete chloroplast genomes of *P. violaris* and related species, including other species from Primulaceae,a phylogenetic tree was constructed. The maximum likelihood (ML) tree was generated using the IQTREE v1.6.7 (Nguyen et al. 2015) with the best selected TVM + F+R4 model. The phylogenetic tree showed that *P. oreodoxa* (NC 050848) is closely related to *P. violaris* and a relationship between *Primula violaris* and 15 Primulaceae species when compared to the outgroup *Androsace bulleyana* NC_034641 (Figure 1). The complete chloroplast genome sequence of *P. violaris* offers the necessary data for phylogenetic studies of Primulaceae. This is hoped that this study can help to solve the problems of intrageneric and interspecific phylogeny in *Primula*.

**Figure 1.**
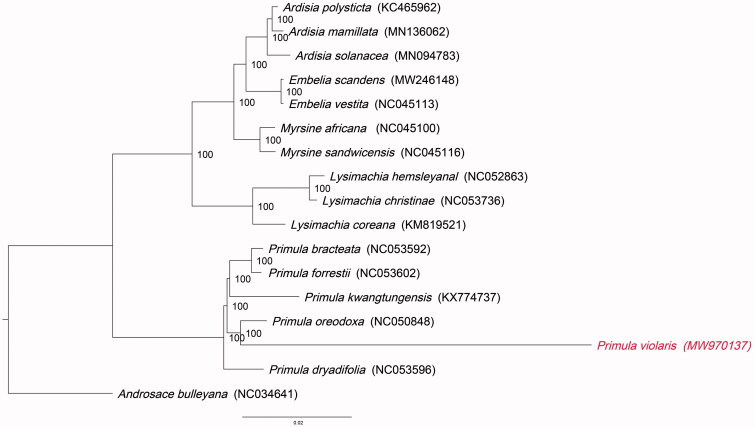
Phylogenetic tree showing the relationship between Primula violaris, 15 Primulales species and outgroup Androsace bulleyana (NC_034641). Phylogenetic tree was constructed based on the complete chloroplast genomes using maximum likelihood (ML) with 1000 bootstrap replicates. Numbers in each the node indicated the bootstrap support values.

## Data Availability

The data that support the findings of this study are openly available in NCBI (https://www.ncbi.nlm.nih.gov) GenBank with the accession number (MW970137). The associated BioProject, SRA, and BioSample numbers are PRJNA723146, SRR14279030 and SAMN18805927 respectively.
